# Enhancing infant pain assessment and treatment: investigating barriers, facilitators, and implementation outcomes with the ImPaC Resource

**DOI:** 10.1186/s43058-026-00856-8

**Published:** 2026-01-10

**Authors:** Mariana Bueno, Kate Pearson, Melanie A. Barwick, Marsha Campbell-Yeo, Christine Chambers, Carole Estabrooks, Rachel Flynn, Sharyn Gibbins, Denise Harrison, Wanrudee Isaranuwatchai, Sylvie LeMay, Melanie Noel, Jennifer Stinson, Anne Synnes, Charles Victor, Janet Yamada, Shirine Riahi, Bonnie Stevens

**Affiliations:** 1 Lawrence Bloomberg Faculty of Nursing, University of Toronto, 155 College Street, Suite 232, Toronto, Ontario M5T 1P8 Canada; 2 The Hospital for Sick Children, Toronto, Ontario Canada; 3 The Hospital for Sick Children, Research Institute, Child Health and Evaluative Sciences, Toronto, Ontario Canada; 4 Faculty of Medicine, Dalla Lana School of Public Health and IHPME, University of Toronto, PsychiatryToronto, Ontario Canada; 5 School of Nursing, Faculty of Health, and Departments of Pediatrics and Psychology & Neuroscience, Dalhousie University, Halifax, Nova Scotia Canada; 6 IWK Health Centre, Centre for Pediatric Pain Research, Halifax, Nova Scotia Canada; 7 Departments of Psychology & Neuroscience and Pediatrics, Dalhousie University, Halifax, Nova Scotia Canada; 8 Faculty of Nursing, University of Alberta, Edmonton Health Clinic Academy, Edmonton, Alberta Canada; 9 School of Nursing and Midwifery, Brookfield Health Sciences Complex, University College Cork, Cork, Ireland; 10 Professional Practice, Trillium Health Partners, Mississauga, Ontario Canada; 11 Department of Nursing, School of Health Sciences, Faculty of Medicine, Dentistry and Health Sciences, University of Melbourne, Melbourne, VIC Australia; 12 Murdoch Children’s Research Institute, Melbourne, Victoria Australia; 13 Faculty of Health Sciences, School of Nursing, University of Ottawa, Ottawa, Ontario Canada; 14 Health Intervention and Technology Assessment Program (HITAP) Foundation, Nonthaburi, Thailand; 15 Faculty of Nursing, and Researcher, Université de Montréal, CHU Sainte-Justine Research Center, TransMedTech Institute and Institut Universitaire en Santé Mentale de Montréal, Montreal, Quebec Canada; 16 Department of Psychology, University of Calgary, Calgary, Alberta Canada; 17 Division of Neonatology, BC Children’s Hospital Research Institute, BC Women’s Hospital, Vancouver, BC Canada; 18 Faculty of Medicine, University of British Columbia, Vancouver, British Columbia Canada; 19 Institute for Clinical Evaluative Sciences (ICES), Management and Evaluation, University of Toronto, The Institute of Health Policy, Toronto, Ontario Canada; 20 Toronto Metropolitan University, Cockwell School of Nursing, DaphneToronto, Ontario Canada; 21 Faculty of Nursing & Faculties of Medicine and Dentistry, University of Toronto, Lawrence Bloomberg, Toronto, Ontario Canada; 22 Management and Evaluation, Institute of Health Policy, Toronto, Canada

**Keywords:** Implementation, Implementation Determinants, Implementation Outcomes, Consolidated Framework for Implementation Research, Infant, Pain

## Abstract

**Introduction:**

The Implementation of Infant Pain Practice Change (ImPaC) Resource is a 7-step, multifaceted, web-based implementation strategy to improve pain assessment and treatment in Neonatal Intensive Care Units (NICUs). We explored facilitators and barriers to implementing ImPaC and their relationship to implementation outcomes.

**Method:**

A hybrid type 1 effectiveness-implementation study was conducted using a cluster randomized controlled trial (reported elsewhere) and a mixed-method exploratory study design. Level 2 and 3 Canadian NICUs with >15 beds were invited to participate and were randomized to intervention (INT, n=12) or usual care (UC, n=11) groups. INT NICUs recruited a change team who accessed ImPaC for 6 months; UC NICUs were waitlisted for 6 months and then offered ImPaC. Focus groups were conducted with all change teams following ImPaC completion. The Consolidated Framework for Implementation Research (CFIR) guided interview questions and analyses. Professionally transcribed interview data were coded and analysed using directed content analysis. Valence (+/-) and strength (–2, –1, 0, +1, +2) were assigned for each CFIR construct/subconstruct. Inductive codes were identified. Relationships between CFIR constructs/subconstructs and ImPaC implementation outcomes (feasibility and fidelity) were determined.

**Results:**

83 NICU change team members (median 4/site) participated in focus groups; 1,105 discrete codes relating to 31 CFIR constructs/subconstructs were identified. The most frequent facilitator constructs were *Design Quality and Packaging*, *Compatibility*, *Available Resources, Champions, Implementation Climate*, and *Engaging Key Stakeholders*. *Complexity* and *Reflecting and Evaluating* were salient in 21 transcripts, and *Patient Needs and Resources* was identified in 20 NICUs. *Available Resources* and *Relative Priority* were barriers. A positive association existed between the feasibility of implementing ImPaC and *Engaging Key Stakeholders* (0.46, p=0.041), *Champions* (0.82, p=0.001), *Relative Priority* (0.75, p=0.001) and *Networks and Communication* (0.60, p=0.023). There was a positive relationship between *Engaging Key Stakeholders* (0.42, p=0.048), *Relative Priority* (0.85, p=0.002), *Patient Needs and Resources* (0.46, p=0.049) and Fidelity.

**Conclusion:**

Site-specific tailoring to enhance facilitators (e.g., champions, implementation climate) and mitigate local barriers (e.g., resources, relative priority) will provide a viable influence on optimizing implementation outcomes.

**Supplementary Information:**

The online version contains supplementary material available at 10.1186/s43058-026-00856-8.

Contributions to the literature

- There is a lack of evidence-based and accessible implementation strategies to improve pain practices across neonatal care settings.
- The Implementation of Infant Pain Practice Change (ImPaC) Resource effectively improved pain practices while being successfully implemented in a hybrid type 1 study.
- The most frequent facilitators (using CFIR) were *Design Quality and Packaging*, *Compatibility*, *Available Resources, Champions, Implementation Climate*, and *Engaging Key Stakeholders*. *Available Resources* and *Relative Priority* were barriers.
- Identifying barriers and facilitators to implementing ImPaC will enable tailoring the intervention and its implementation to the local level to promote clinical and implementation outcomes.

## Background

Successful implementation of research evidence into practice is a well-documented challenge [1]. Pain in infants is primarily associated with necessary clinical procedures and is frequently undertreated, unrecognised, or poorly managed [2, 3]. Persistent and untreated pain in early life can result in significant and enduring consequences that extend into adulthood, including continued chronic pain, disability, and distress [2]. This undertreatment of pain need not continue given the available tools, expertise, and evidence to prevent and treat pain in infants [2]. Educational materials, readiness for change questionnaires, audits, and intervention or quality improvement (QI) templates are implementation tools that have been reported to effectively change outcomes in single hospital Neonatal Intensive Care Units (NICUs) [4–8]. However, we are unaware of any comprehensive and accessible implementation strategies to improve pain assessment and treatment more globally across infants and settings.

The *Implementation of Infant Pain Practice Change* (ImPaC*) Resource* (Table 1) is a 7-step, multifaceted, web-based implementation strategy designed for healthcare providers to improve assessment and treatment of procedural pain in NICUs. ImPaC is comprised of strategies such as champions (Table 1, Step1), readiness assessment (Table 1, Step 2), audit and feedback (Table 1, Steps 3 and 6), a library of up-to-date and evidence-based knowledge translation tools on pain assessment and management (Table 1, Step 4) that enables champions to foster a collaborative learning environment while implementing these tools in their clinical settings (Table 1, Step 5), and finally a plan for next steps (Table 1, Step 7). ImPaC builds on the “Evidence-based Practice for Improving Quality” (EPIQ) for improving pain practices in children, which is comprised of high-quality evidence and QI methods using interactive implementation strategies. Improved pain practices and clinical outcomes were achieved in 16 Canadian hospital units that received the EPIQ intervention (INT) compared to 16 units that continued with usual care (UC) [10]. However, EPIQ was not web-based and thus was not always considered feasible or cost-effective [11], and results were only partially sustained over 12–36 months [10, 12]. ImPaC was developed using a user-centered process to address identified shortcomings and was deemed feasible, easy to navigate, engaging, intuitive, and comprehensive from a series of usability tests [13].

**Table 1:** ImPaC Resource steps [9]

Step	Description of ImPaC Steps	Estimated Time to Complete
1	Complete a checklist on the characteristics and strengths of a small team of 3–5 healthcare professionals responsible for implementing a practice change on the unit.	4 weeks
2	Complete the ImPaC readiness for change survey and review your individual or team results.	
3	Using the brief audit tool, perform about 10 infant chart audits to identify the unit’s current pain assessment and treatment practices.	
4	a. Based on the audit results, select a pain assessment measure or pain treatment intervention that will be the focus for practice change. b. Review the evidence briefs. Consider measures or interventions that are the most feasible, relevant, and important for the infants, staff, and organization at this time. c. Create an aim statement and specify the percentage of change the team is aiming to achieve, and the time frame to achieve it. For example: We aim to increase pain assessment with the NIPs tool from 20% to 40% over the next 8 weeks.	2–3 months per cycle
5	a. Select the knowledge translation (implementation) tools (e.g., mini presentations, stickers, screen savers) to help implement the pain assessment or treatment change into practice. b. Complete the activity planner to develop a detailed implementation plan.	
6	As in Step 3, use the audit tool to perform at least 10 infant charts audits following the practice change implementation period. Compare the audit results with the results generated in Step 3 to see if there is improvement.	
7	Evaluate the pain practice change and the implementation process. Repeat Steps 4–7 (cycle) until the desired practice change is achieved.	

In a hybrid type 1 effectiveness-implementation study, 23 level 2 and 3 NICUs across Canada were enrolled in a cluster randomized controlled trial (cRCT) and randomized to the INT or UC groups [9]. In the INT group, the average number of procedures/infant/day was less, the proportion of procedures associated with pain assessment and pain treatment was greater, and pain intensity scores were lower. Although clinical outcomes were significantly improved, only 14/23 (60.9%) NICUs implemented ImPaC as intended for at least one complete 7-step cycle. NICUs spent an average of 10.18 (±4.36) hours implementing ImPaC over six months. More time spent using ImPaC with fidelity was associated with fewer painful procedures/infants/days and improved pain assessment and treatment strategies. Although the INT group outcomes were significantly better than the UC group, the pain assessment and treatment provision were still relatively low [9].

ImPaC is a multifaceted, novel implementation strategy that has the potential to bridge the knowledge-to-practice gap at the individual (e.g., by introducing interactive education and QI interventions and templates) and system (e.g., by promoting the use of champions/change teams) levels while determining change readiness and fostering a positive unit culture.

Understanding implementation barriers or facilitators and how they influence implementation outcomes is crucial to ensuring meaningful and sustainable pain practices.

### Theoretical framework

The Consolidated Framework for Implementation Research (CFIR 1.0) [14] provided the conceptual basis for the study. CFIR is an implementation science (IS) determinant framework comprised of 39 constructs/subconstructs across five domains: (1) Intervention (Innovation) Characteristics, (2) Inner Setting, (3) Outer Setting, (4) Characteristics of Individuals, and (5) Process. CFIR guided the development of interview questions, coding and analyses.

## Methods

### Aim

We aimed to: (1) identify barriers and facilitators to implementing ImPaC in Canadian NICUs; and (2) explore the relationship between determinants and implementation outcomes (feasibility and fidelity).

### Design and setting

This mixed-methods exploratory study was nested in a hybrid type 1 effectiveness-implementation study and a cRCT [9, 15]. Level 2 or 3 NICUs in pediatric or general hospitals across Canada were invited to participate. NICUs were eligible if they: (1) had a minimum of 15 beds; and (2) agreed to be engaged in the study for up to 24 months. NICUs were randomized to INT (n=12) or UC (n=11) groups using a computer-generated random allocation sequence (randomize.net).

### Participants

Three to five individuals in each participating NICU were invited to join the ImPaC change team (CT) if they were a healthcare professional (HCP), English speaking, had ≥3 years of NICU experience, had flexibility and time within their role to engage in the study, and had clinical leadership experience (e.g., advanced practice or clinical education role). A local research coordinator obtained research consent. All CT members were trained and given access to ImPaC for 6 months. The INT NICUs received access to ImPaC immediately after randomization, while the UC NICUs were waitlisted for 6 months and then offered access to ImPaC. More details on participants recruitment and training are described elsewhere [9, 15]. Upon completion of the intervention for both the INT and UC groups, CT members were invited to participate in focus group (FG) interviews.

### Data collection

Researchers (MB, BS) with qualitative research expertise conducted virtual FG interviews following the completion of the ImPaC intervention. Participants had no prior relationship with the interviewers. Interviewers used a CFIR-informed, semi-structured interview guide. Interviews were digitally recorded, de-identified, transcribed verbatim by a professional transcriptionist, and reviewed for accuracy. Each transcript represented a FG interview with a single NICU. Implementation outcomes were operationalized as user activity captured from the “back-end” of ImPaC. Feasibility (the number of hours the CT used ImPaC within the NICU) was assessed as an indicator of ImPaC’s ease of use in practice. Intervention fidelity was assessed as the degree to which ImPaC was implemented as intended (e.g., completing the seven steps of the change cycle).

### Data analysis

#### Qualitative analysis

We undertook a deductive-inductive hybrid thematic analysis approach [16], allowing for structured analysis of data with a well-established and rigorously conceptual framework – CFIR guided a directed (deductive) content analysis – and for codes generated from the data itself, through inductive analysis. Our deductive approach was modelled from previous work done with the CFIR framework [17] and is described in detail in Additional file 1.

First, two research coordinators (KP and MR) familiarized themselves with the CFIR framework, construct/subconstruct definitions, and inclusion and exclusion criteria.

Second, the research coordinators and an IS researcher (MB) reviewed the CFIR framework and used the CFIR Codebook template [18] to create a coding dictionary in MAXQDA. Code memos were added detailing definitions and inclusion and exclusion criteria. The code memos were further developed using an iterative process to document how the CFIR codebook was operationalized to the study context [19]. Five subconstructs were not considered relevant to the study. They were not included in the coding dictionary (i.e., *Trialability, Peer Pressure, Organizational Incentives and Rewards, Formally Appointed Internal Implementation Leaders,* and *External Change Agents*), leaving the coding dictionary with 36 CFIR constructs and subconstructs. Next, the three analysists worked together to code the two transcripts line-by-line. Following consensus, segments were coded using a single CFIR code. Segments were inductively coded whenever the content was considered relevant to the study but did not align with CFIR. Inductive codes were created based on group consensus and used independently or combined with a CFIR code. The two research coordinators independently coded a third and fourth transcript. Then, all analysts compared CFIR codes, discussed any disagreements and, by consensus, reached decisions while generating a final coded transcript. The remaining transcripts were independently coded by one of the analysts and then reviewed by the others.

Strength and valence ratings were assigned to each construct based on a deliberate consensus approach in each transcript. These ratings were based on the content of the coded segments in relation to the negative or positive influence of the construct and/or subconstruct in the implementation process [14, 17]. They reflected the valence (positive [+] or negative [-] influence) and strength (from −2 to +2) of coded constructs and subconstructs in each transcript.

Summary reports for each transcript listed the ratings for each CFIR construct with at least one supporting segment. When all constructs and/or subconstructs were rated, they were compared across summaries (i.e., across sites) to help ensure consistency [17].

#### Quantitative analysis

Quantitative metrics were objectively determined from ImPaC on time spent using the Resource and the number of cycles or steps completed. Means and standard deviations were used to summarize continuous implementation outcomes: (a) feasibility (time spent by the change team using ImPaC in practice setting, calculated through automatic capture of the website’s back-end data metrics as users signed in/out) and (b) fidelity (adherence to completing the change team’s progression through steps or cycles). Spearman’s correlation was used to assess the association between each construct and the feasibility and fidelity outcomes. A p-value of less than 0.05 was considered statistically significant.

### Reporting guidelines

This manuscript was prepared following the Standards for Reporting Implementation Studies (StaRI) [20].

## Results

### Facilitators and barriers to implementing ImPaC

We enrolled 107 CT participants and conducted 23 FG with 83/107 individuals (median 4 participant/site, range 1 to 7) from all participating NICUs. Interviews were conducted between January 21, 2021 and December 2, 2022, and lasted 25–60 minutes (Mean, 45.2, Median 46.5).

Table 2 reports the demographic characteristics and self-reported pain practices of the 107 CT participants. Most were full-time staff nurses with bachelor’s degrees. They were generally very experienced, and all were women. They reported a wide range of frequency of pain assessment and treatment practices as well as barriers and facilitators to implementing the ImPaC Resource and more generally, pain practice change.

**Table 2:** Demographic characteristics and self-reported pain practices

Demographics and pain practices	N (%)
Role	
Staff Nurse	35 (48.0)
Charge Nurse	8 (11.0)
Nurse Educator	12 (16.4)
Nurse Practitioner	5 (6.9)
Clinical Nurse Specialist	1 (1.4)
Physician	1 (1.4)
Other	11 (15.1)
Employment	
Full-time	54 (74.0)
Part-time (0.5 FTE or more)	17 (23.3)
Part-time (Less than 0.5 FTE)	2 (2.7)
Gender	
Female	73 (100.0)
Education	
Diploma/Certificate	15 (20.6)
Bachelors Degree	53 (72.6)
Masters Degree	14 (19.3)
Experience (in years) in current role	
Less than 1 year	5 (6.9)
1 year to 5 years	25 (34.3)
6 years to 10 years	7 (9.6)
More than 10 years	36 (49.3)
Experience (in years) in neonatal/pediatric care	
1 year to 5 years	18 (24.7)
6 years to 10 years	5 (6.9)
More than 10 years	50 (68.5)
Frequency of pain assessments in their clinical practice	
Never	9 (12.3)
Rarely	9 (12.3)
Sometimes	19 (26.0)
Most of the time	16 (21.9)
Always	20 (27.4)
Frequency of pain treatment provision in their clinical practice	
Never	1 (1.4)
Rarely	10 (13.7)
Sometimes	21 (28.8)
Most of the time	24 (32.9)
Always	17 (23.3)

The analysists coded all transcripts, and 1,105 discrete codes relating to 31 CFIR constructs and subconstructs were identified. The most frequent or salient CFIR constructs were *Design Quality and Packaging* and *Compatibility*, coded in all 23 transcripts, followed by *Available Resources, Champions, Implementation Climate,* and *Engaging Key Stakeholders*, emerging in 22 transcripts. *Complexity* and *Reflecting and Evaluation* were evident in 21 transcripts, whereas *Patient Needs and Resources* was salient in 20 transcripts. CFIR constructs *Goals and Feedback, Learning Climate, Individual Identification with the Organization, Readiness for Implementation, and Engaging,* were not evident in any transcripts.

Details on the frequency, valence and strength attributed to each of the 31 constructs coded are presented in Tables 3 and 4. Constructs included in the coding dictionary but not identified as a facilitator or barrier by at least one CT were omitted.

**Table 3:** Frequency of facilitators and barriers from coded transcripts

**CFIR construct/subconstruct* (italic)***	**Barrier (−1,−2)**	**Facilitator (+1, +2)**	**Mixed** **(X)**	**Neutral** **(0)**	**Total**
**Innovation Characteristics**					
Innovation source	0	3	0	0	3
Evidence strength and quality	1	15	1	1	18
Relative advantage	2	2	0	1	5
Adaptability	0	11	1	3	15
Complexity	7	13	1	0	21
Design and quality packaging	1	21	1	0	23
Cost	3	9	0	4	16
**Inner Setting**					
Structural characteristics	3	2	0	2	7
Networks and communication	1	10	1	3	15
Culture	1	3	1	1	6
Implementation climate	7	11	3	1	22
*Tension for change (climate)*	0	4	0	0	4
*Compatibility (climate)*	1	18	0	4	23
*Relative priority (climate)*	14	0	0	2	16
*Leadership engagement (Readiness)*	4	11	1	2	18
*Available resources (Readiness)*	15	4	1	2	22
*Access to knowledge and information (Readiness)*	7	7	0	0	14
**Outer Setting**					
Needs & resources of those served by the organization	0	15	1	4	20
Cosmopolitanism	1	0	0	2	3
External policy and incentives	0	1	0	0	1
**Characteristics of individuals**					
Knowledge and beliefs about the innovation	2	5	1	7	15
Self-efficacy	0	1	0	1	2
Individual stage of change	0	2	0	0	2
Other personal attributes	0	6	0	0	6
**Process**					
Planning	1	4	1	3	9
*Opinion leaders (Engaging)*	0	2	0	0	2
*Champions (Engaging)*	8	5	2	7	22
*Key stakeholders (Engaging)*	1	13	1	7	22
*Innovation participants (Engaging)*	1	3	0	2	6
Executing	5	5	1	6	17
Reflecting and evaluating	1	12	0	8	21
**Inductive code**					
COVID-19	15	0	0	2	17

**Table 4:** Constructs and subconstructs strength and valence, per site

**Site** **Domain, Construct/Subconstruct**		**1**	**2**	**3**	**4**	**5**	**6**	**7**	**8**	**9**	**10**	**11**	**12**	**13**	**14**	**15**	**16**	**17**	**18**	**19**	**20**	**21**	**22**	**23**
**Innovation characteristics - 7/8**	Innovation source	NC	NC	NC	NC	NC	NC	NC	NC	NC	NC	+2	NC	NC	+2	NC	NC	NC	+1	NC	NC	NC	NC	NC
	Evidence strength and quality	NC	+1	+1	0	+2	+1	X	+2	+1*	+2	+2	−1*	NC	+2	NC	NC	+2	+1	+1	+1	+1	NC	+1
	Relative advantage	−1*	NC	NC	NC	+1	−1	NC	NC	NC	0	NC	NC	NC	NC	+1	NC	NC	NC	NC	NC	NC	NC	NC
	Adaptability	X	+1	+1	+1	NC	NC	+1	+1	0	+1	NC	NC	NC	NC	+1	NC	+1	0	+1	+1	0	+1	NC
	Complexity	−1*	X	+1	+1	+1	−2	NC	+2	+1	−1	+1	+1	−1*	−1*	+1	NC	+1	+1	+1	+1*	−1*	−1*	+1
	Design and quality packaging	+1*	+2	+1	+1	+2	+1*	+1	+2	+1*	+1	+1	+1*	+1*	+2	+1*	−1	+1	+1	+1	+2	+1*	X	+1
	Cost	0	NC	+1	0	+2	−1	NC	0	−1	+1	NC	NC	+1	0	−1	NC	+1	+1*	+1	0	+1	+1	0
**Outer setting - 3/4**	Needs & resources of those served by the organization	NC	+1	NC	+1	+1	+1	+1	+1	+2	0	+2	+1	0	+1	X	0	NC	+1	+1	0	+1	+1	+1
	Cosmopolitanism	NC	NC	NC	NC	NC	0	NC	NC	NC	NC	NC	NC	NC	NC	NC	NC	−1	NC	NC	0	NC	NC	NC
	External policy and incentives	NC	NC	NC	NC	NC	NC	NC	NC	+1	NC	NC	NC	NC	NC	NC	NC	NC	NC	NC	NC	NC	NC	NC
**Inner setting - 13/14**	Structural characteristics	NC	NC	−1	NC	NC	+1	NC	NC	NC	NC	+1	NC	0	NC	NC	0	NC	NC	NC	NC	−1	NC	−1
	Networks and communication	NC	+1	+1	NC	+1	+1	0	NC	NC	0	+1	NC	NC	+1	+1	NC	NC	+1	−1	+1	+1	0	X
	Culture	NC	+1	NC	NC	NC	+1*	NC	NC	NC	+1	0	NC	NC	NC	X	NC	NC	NC	NC	NC	−1	NC	NC
	Implementation climate	+1	+2	−1	+1	−1	+1*	−1*	0	−1	X	+1	−1	−2	−1*	X	NC	+1	+1	+1	+2	X	+1	+1
	*Tension for change (climate)*	NC	NC	NC	NC	NC	+1	+1	NC	NC	+1	NC	NC	NC	NC	NC	NC	NC	NC	+1	NC	NC	NC	NC
	*Compatibility (climate)*	0	+2	+1*	0	+2	+1	+2	+1	+1	0	+1	+1	+1	+1	+1	+1	+1	+1	+1	−1	+2	0	+1
	*Relative priority (climate)*	0	NC	NC	−2	−2	−1	NC	−1	−2	−1	−1	NC	−1	−1	NC	−2	−1	−1	NC	−1	0	−1	NC
	Goals and feedback	NC	NC	NC	NC	NC	NC	NC	NC	NC	NC	NC	NC	NC	NC	NC	NC	NC	NC	NC	NC	NC	NC	NC
	Learning climate	NC	NC	NC	NC	NC	NC	NC	NC	NC	NC	NC	NC	NC	NC	NC	NC	NC	NC	NC	NC	NC	NC	NC
	Readiness for implementation	NC	NC	NC	NC	NC	NC	NC	NC	NC	NC	NC	NC	NC	NC	NC	NC	NC	NC	NC	NC	NC	NC	NC
	*Leadership engagement (readiness)*	NC	+1	NC	+1	+1	+1	+1	+1	−1	NC	+2	+1	0	−1	−1	X	NC	0	+1	NC	+1	−1	+1
	*Available resources (readiness)*	−1	+1	+1	−2	X	−2	+2*	−2	0	−1	0	−1	−1	−2	−1	−2	−1	+1	−1	NC	−1	−2	−1
	*Access to knowledge and information (readiness)*	−1	+1	+1	NC	NC	−1	NC	NC	−1	+1	+1	NC	−1*	+1	+1	−1	NC	NC	NC	−1	−1	+1	NC
**Characteristics of individuals - 5/5**	Knowledge and beliefs about the innovation	+1	NC	0	0	NC	0	NC	+1	0	NC	+1	NC	NC	NC	−1	0	0	+1	0	X	+1	−1	NC
	Self-efficacy	NC	NC	NC	NC	NC	NC	NC	NC	NC	NC	+1	NC	NC	NC	NC	NC	NC	NC	NC	NC	NC	NC	0
	Individual stage of change	NC	NC	NC	NC	NC	NC	NC	NC	NC	+1	NC	NC	NC	NC	NC	NC	NC	NC	NC	NC	NC	NC	+1
	Individual identification with organization	NC	NC	NC	NC	NC	NC	NC	NC	NC	NC	NC	NC	NC	NC	NC	NC	NC	NC	NC	NC	NC	NC	NC
	Other personal attributes	NC	NC	NC	NC	NC	NC	+1	NC	NC	NC	NC	NC	+1	NC	+1	NC	NC	NC	NC	NC	+1	+1	+1
**Process - 8/10**	Planning	NC	0	NC	+1	NC	NC	NC	+1	+1	NC	NC	+1	NC	0	NC	−1	NC	0	NC	NC	X	NC	NC
	Engaging	NC	NC	NC	NC	NC	NC	NC	NC	NC	NC	NC	NC	NC	NC	NC	NC	NC	NC	NC	NC	NC	NC	NC
	*Opinion leaders*	NC	NC	NC	NC	NC	NC	NC	NC	NC	NC	+2	NC	NC	NC	NC	NC	NC	NC	NC	NC	+1	NC	NC
	*Champions*	NC	+1	0	−1	−1	0	−1	0	−1	X	+1	−1*	−1	+1	0	−2	X	0	0	0	+1	−2	+1
	*Key stakeholders*	NC	+2	+1	0	0	0	+1*	+1	+2	X	+2	+1	−2	+2	+1*	0	0	0	+2	0	+2	+2	+1
	*Innovation participants*	NC	NC	NC	+1	NC	NC	NC	NC	NC	−1*	+1	NC	NC	+2	NC	NC	NC	NC	NC	NC	0	NC	0
	Executing	0	NC	+1	0	NC	NC	0	−1	−1	+2	+1	−1	−1	0	NC	0	0	NC	+1	NC	X	−1	+1
	Reflecting and evaluating	0	+1	+1	+1*	0	+1	NC	+1	+1	+1	0	0	−1	+1	0	NC	0	+1	+1	+1*	+1	0	0
	Barrier - COVID	−2	−1	−1	−2	−2	−2	−2	−1	0	−1	0	−1	−1	−1	−1	−2	NC	NC	NC	NC	NC	−1	−1

*NC* Not coded

### Intervention characteristics

The perceived excellence in how the ImPaC is bundled, presented, and assembled [*Design Quality and Packaging*] was among the most salient implementation determinants, predominantly indicated as a facilitator.

“*It was very functional. It was very easy for us to use, and like I said before, the information is straight and to the point, so there’s not a lot of extra wording that people would just skim over or not even read at all. It was very user-friendly*.” (Site W)

The library of KT tools (e.g., evidence briefs, PowerPoint presentations, posters, stickers, lanyards, etc., in Step 5 of ImPaC) was considered user-friendly, easy to navigate, well-designed, and organized.

“*Going into an environment that was already incredibly overwhelming at this time, the resources we were able to put forth to the staff were simple, they were concise, they were easy to follow, and it was not another level of information overload*.” (Site E)

*Evidence Strength and Quality*– the stakeholders’ perceived quality and validity of evidence supporting the belief that the intervention will have the desired outcomes - was also a facilitator for most of the CTs:

“*It’s a lot of incredibly valuable information available at our fingertips. There’s no need to reinvent the wheel. It’s information and research that’s done, so why not use it, and it’s available to us. I think that alone is incredibly beneficial to be able to roll out so much in-depth and precise information on something that we clearly are supportive and strive to improve on within our unit*.” (Site E)

The *Intervention Source* was described as another facilitator, with CTs recognizing ImPaC as developed by a reputable, credible group “*for nurses by nurses*”. (Site K)

Although the majority of the NICUs did not perceive ImPaC as a complex intervention [*Complexity*], some CTs felt that transitioning from one step to the next was challenging, especially while completing the pain practice audits in Steps 3 and 6:

“*I think it set us up pretty well for the following steps. I wouldn’t say that there was anything else that was missing. Again like having to go through each step one by one was helpful to get a sense of what we’re doing now. Okay we’ve completed that and now it’s the next one. So that and everything leading up to the actual implementation was fine*.” (Site H)

Participants stated that ImPaC could be adapted or tailored to meet the NICUs’ needs [*Adaptability*], such as training newly hired clinicians and staff from units that eventually cared for the infants (e.g., pediatric units). One participant mentioned that ImPaC could be adapted to other practice changes as “*it gives a good outline and a step-by-step process of how to carry out a change*” (Site C).

The *Cost* of implementing ImPaC was considered minimal (e.g., using hospital printers to print posters). Still, operating costs would need to be considered by CTs when implementing the KT tools in their setting. A small budget was provided for teams to carry out these activities in the research study:

“*And I think, too, knowing that we have the budget, we were able to choose printable pieces like the posters and the lanyards, and if we didn’t have that budget, we would have considered more electronic like the screensavers or emails or other presentations*.” (Site F)

*Relative Advantage,* the perception of the advantage of implementing the intervention versus an alternative solution, was a facilitator. CTs provided the team with “*a solid place to go from*” (Site E) and assisted with keeping up with timelines and the next steps. Conversely, others stated that ImPaC did not provide advantages to previous practice changes. Sites were comfortable using an externally developed tool for practice change in their units.

### Inner setting

*Compatibility* (the fit between meaning and values attached to the intervention by involved individuals, how those align with individuals’ norms, values, and perceived risks and needs, and how the intervention fits with existing workflows and systems) was a facilitator.

“*I think that the tools actually were a reinforcement of the…what we have…the education that we have regarding the sucrose and it was just a really good reminder and it's nice to have some other education other than our voices for the staff to have something other than us to present what is out there*.” (Site S)

The CTs described a favourable *Implementation Climate* characterized by receptiveness, interest and responsiveness to the innovation and the implementation process. A positive implementation climate was considered a facilitator by most with staff “(…) *coming and saying can you tell me more about it.*” (Site Q)

“*So in our unit it is very high acuity and very kind of fast paced…staffed with those exact personalities. And so I think everyone is really game for implementing change and very quick to adapt. So in that sense it was very feasible and I would say that to me it not only speaks to the tool but the people that I work with*.” (Site T)

Some units indicated that “(…) *people are just getting really burnt out with seeing here’s another change*.” (Site M). In addition, the staff perceived that there were “(…) *more databases to fill out, more paperwork to fill out*” (Site I) because of the need to improve documentation of pain practices to assess practice change.

The support of mid-level leaders (e.g., clinical leaders and educators) was also evident [*Leadership Engagement*]. Several teams highlighted their commitment, involvement, and accountability as facilitative. Participants indicated that a “*big change*” (Site G) would not be possible without leadership engagement and stressed the importance of their active involvement with the CT members in discussing and making decisions on their implementation processes.

“*Yes, I definitely agree that having that leadership and upper management support is vital in terms of implementing the resource or really any practice change amongst the unit.*” (Site K)

Local formal and informal *Networks and Communications* facilitated ImPaC within and across boundaries in the inner setting. Huddles, educational sessions, and educational boards as regular practices in the units favoured the implementation activities:

“*We discussed it every morning, too, in the morning huddle. So every week we would have new staff…residents, NPs, students, med students and, so then they would become aware of it and what our aim was and what our goals were*.” (Site U)

Some teams indicated that they worked with IT departments to adjust their medical charting system and included relevant information for their practice change, highlighting the importance of communication for the CT's functioning.

Although all participants interacted with ImPaC and reported completing at least some of the seven core steps, *Relative Priority* was a barrier for some. One of the teams acknowledged that “(…) *there always seems to be competing priorities and I don’t know if you will ever get to the point where there is no competing priorities. I think that’s just the nature of our environment*.” (Site U) The COVID-19 pandemic, other quality improvement initiatives, implementation of electronic charting systems, staff turnover, staff shortages, and seasonal increased hospitalizations were some of the competing priorities mentioned:

“*But I think you also caught us in a time when we were also kind of getting…the Covid piece was slowly sorting itself out and now obviously there’s other pressures within the hospital in terms of pediatric viral illnesses that we’re definitely seeing trickling down to the NICU so I would say we’re just during a time of…I don’t want to say, like, high stress but lots of things happening, and I think, yes, it was feasible but perhaps wasn’t a super high priority for some of the bedside frontline staff. But I don’t think it impacted their ability to implement some of the tools.*” (Site T)

The *Available Resources* dedicated to implementation and ongoing operations, including money, training, education, physical space, and time, were considered insufficient for ImPaC implementation in many sites, especially concerning time to work on their practice change.

“*We were understaffed. My assignments were so heavy, I barely got to my breaks. I’m barely getting to my breaks at all. So, just even having five minutes to kind of come back and look it was just…it was too much and it wasn’t a priority*.” (Site P)

### Outer setting

Within the outer setting domain, the most salient facilitator was the needs of the neonates and their families [*Patient Needs and Resources*]. Participants indicated that ImPaC brought awareness about pain assessment and treatment, making neonates and their families more comfortable in the NICU.

“*And people can get creative and realize that it is true…when it makes everyone’s…you feel your job easier, the baby is more comfortable, the parents are more comfortable…things will be more smooth and people will start to, maybe, be more creative and have more ideas of implementing the skin-to-skin or the breastfeeding but this is the kind of first step I think into that*.” (Site K)

### Characteristics of individuals

Participants considered themselves familiar with neonatal and infant pain knowledge and QI practices. Their *Knowledge and Beliefs* facilitated their role in the implementation of ImPaC:

“*Because a lot of us as people with leadership experience from different disciplines and many of us were on pain committee to begin with were used to these PDSA cycle and with the understanding of our group and what new practice implementation looks like…so I think we just dove headfirst into it*.” (Site H)

Other *Personal Attributes* such as diversity in personal background, roles (e.g., clinicians, educators, NPs), clinical and technological expertise, and knowledge perspective contributed to implementation:

“*Yes I think it was helpful to have a variety of people with different backgrounds and different experience levels and what not.*” (Site M)

### Process

In several interviews, the staff members engaged in implementation activities described the CTs as facilitators [*Engaging Key Stakeholders*]. CTs used different strategies to engage staff, such as educational sessions, huddles, electronic reminders and “*spontaneous discussions that happened at the bedside as well amongst the staff.*” (Site B) Participants perceived that staff were receptive to the practice change, disseminating and implementing what they learned from the CTs at the bedside.

*Reflecting and Evaluating* on implementation progress and quality was also a facilitator. The audit tool and the pre- and post-intervention reports (Steps 3 and 6) enabled the CTs to reflect on their clinical and implementation outcomes and the results achieved and plan for their next steps. Some of the participants indicated they shared the results with staff, especially when their goals were achieved:

“*So I think people like to see numbers when they rise. So, showing them where we started at and what we are at now…people’s eyes were opened when we said this is what we achieved the last six months, and they are like, what? Wow. So I think people like that. And I like it, every now and then. So, I think if we have good numbers and we can show them that there’s been a change in the practice that has been reflected in our numbers, I think that would be a plus, every now and then*.” (Site J)

*Executing* the implementation according to plan was considered a facilitator for CTs. ImPaC “(…) *is quite reasonable. I was very impressed at how fast and we quickly moved to a positive outcome*.” (Site S) However, other CTs “(…) *didn’t get all the steps done. We got to probably step four or step five and then it just stopped*.” (Site P) One site indicated the seven-step process was too long and was a barrier to implementing their practice change. Some CT champions felt overwhelmed trying to implement ImPaC:

“*Because we are a small group and it seems the same people are implementing the same projects, and so, it’s a bit of, also, overload, especially during Covid. So, I think you’re also/we need to mindful of how many champions we have when we are actually doing these types of programs because it’s almost overload for those that are rolling them out or ensuring that we are doing what we are supposed to be doing*.” (Site V)

In addition, as *Champions*, CT members described how they needed to adapt their approach:

“(…) *We had to muster up energy. We…not only at the bedside but also with our colleagues. That was really difficult because we were coming with all this energy, but then for it not to be reciprocated was…we had to adapt. We had to recognize that, and I think that in and of itself was a huge challenge*.” (Site G)

### Relationship between implementation determinants and implementation outcomes

Several barriers and facilitators were associated with implementation outcomes (feasibility and fidelity) (Table 5).

**Table 5:** Correlation between constructs and subconstructs and implementation outcomes

**Construct/Subconstruct**	**Correlation feasibility**	**p-value**	**Correlation with fidelity**	**p-value**
Innovation Characteristics				
Innovation source	-	-	−0.98	0.12
Evidence strength and quality	0.06	0.81	0.04	0.86
Relative advantage	−0.50	0.4	0	0.99
Adaptability	−0.41	0.15	0.13	0.65
Complexity	−0.39	0.21	−0.12	0.56
Design and quality packaging	0.35	0.12	0.25	0.26
Cost	−0.14	0.59	0.33	0.18
Outer Setting				
Patient needs and resources	0.19	0.46	0.46	0.049*
Cosmopolitanism	0.95	0.21	0.99	0.084
External policy and incentives	-	-	-	-
Inner Setting				
Structural characteristics	−0.32	0.49	−0.18	0.7
Networks and communication	0.60	0.023*	0.004	0.99
Culture	−0.77	0.12	0.31	0.61
Implementation climate	0.20	0.43	0.15	0.63
Tension for change	**-**	**-**	-	-
Compatibility	0.25	0.26	−0.1	0.64
Relative priority	0.75	0.001*	0.85	0.002*
Goals and feedback	**-**	**-**	-	-
Learning climate	**-**	**-**	-	-
Readiness for implementation	**-**	**-**	-	-
Leadership engagement	0.06	0.83	0.28	0.28
Available resources	0.23	0.33	0.04	0.86
Access to knowledge and information	0.27	0.36	−0.9	0.75
Characteristics of individuals				
Knowledge and beliefs about the innovation	0.43	0.12	0.34	0.24
Self-efficacy	**-**	**-**	**-**	**-**
Individual stage of change	**-**	**-**	**-**	**-**
Individual identification with organization	**-**	**-**	**-**	**-**
Other personal attributes	**-**	**-**	**-**	**-**
Process				
Planning	−0.02	0.97	0.13	0.75
Engaging	-	-	-	-
Opinion leaders	-	-	-	-
Champions	0.82	<0.001*	0.13	0.59
key stakeholders	0.46	0.041*	0.42	0.048*
Innovation participants	−0.10	0.85	−0.63	0.18
Executing	0.31	0.27	0.26	0.32
Reflecting and evaluating	0.33	0.16	0.19	0.41
Inductive code				
COVID-19	0.42	0.095	−0.31	0.21

*Denotes statistically significant correlation

There was an association between *implementation recipients* and feasibility (0.46, p=0.041) and fidelity (0.42, p=0.048). There was also a significant relationship between *champions* and time using ImPaC (0.82, p=0.001).

CT members described how staff perceived ImPaC:

“*They were really receptive when we said it was just a quick minute and a half long video they were like okay…that’s something I’m willing to watch. So, I think that was really helpful and you know…I noticed lots of people watching it. I think that people…they didn’t feel like it was redundant or threatening*.” (Site N)

Staff’s positive receptivity might have influenced the time CTs were able to use ImPaC in the NICU:

“*(…) I feel like that’s one of our most successful strategies, in terms of putting the information out there, and along with the videos and posters, we have some evidence briefings. Some of the…some of the evidence briefs from within the resource that we’ve printed and tried to make them readily accessible within the unit and in high traffic areas and like we had mentioned with the little competition that we’re just trying to raise awareness and promote and encourage everyone to document and continue doing these pain management…nonpharmacological pain management techniques*.” (Site K)

Some CT members indicated that their role as *Champions* facilitated the implementation of ImPaC.

I felt *“(…) encouraged to be in a group so I didn’t have to take it on myself. So it just…I felt like the website encouraged that interaction as a team. You know? And using it.*” (Site W) However, other CTs disagreed, because *“(…) none of us really ever ran on the same schedule other than a couple of people. And…just finding time in the day to really sit down and go through anything was quite challenging”.* (Site M)

*Networks and Communications* were significantly correlated with the feasibility of implementing ImPaC (0.60, *p*=0.023). CTs engaged in implementation activities through opportunities available at their unit, which mainly were perceived as facilitators:

“*We discussed it every morning too, in the morning huddle. So every week we would have new staff…residents, NPs, students, med students, and so, then they would become aware of it and what our aim was and what our goals were*.” (Site U)

There was also a significant relationship between *Relative Priority* and feasibility (0.75, np=0.001) and fidelity (0.85, p=0.002). However, *Relative Priority* was also considered a barrier that often precluded them from spending time on the website.

“*I feel like everyone was already, like, super stressed about everything. They were stressed about COVID. They were stressed about EPIC. We happened to be probably the busiest we’ve been in a long time too during this period, as well.*” (Site D)

“*And I think it was just like timing from our site. We were…we were extremely, extremely busy in the last few months, and we had management change, and we had staffing…serious staffing issues. So, then, just being at work was…is getting the work done*” (Site I).

Finally, a significant relationship existed between *Patient Needs and Resources* and fidelity (0.46, p=0.049):

“*I think we’re very good for advocating for our patients and trying to ensure that they are comfortable. So staff bought into the value of it, so I think that made it more successful*.” (Site I)

## Discussion

### Summary of findings and comparisons with other studies

This study aimed to explore barriers and facilitators of implementing ImPaC, a multifaceted novel implementation strategy, across 23 Canadian NICUs and to determine relationships between implementation determinants and implementation outcomes. The most frequent facilitators were within the *Innovation Domain*.

The iterative integrated implementation approach, bringing developers and users together, to develop and refine ImPaC resulted in its’ *Design and Quality Packaging* being identified as a facilitator by almost all the CTs. Also, using the web-based format allows for greater accessibility, relative ease to sustain and modify once developed, and automation and interactive capabilities [21].

In addition, the perceived *Evidence Strength and Quality* of ImPaC was highly salient and a key facilitator for practice change. Participants perceived the pain assessment and treatment tools as being of high quality and valid. The readily available library of resources on pain assessment and treatment comprised up-to-date, high-quality, synthesized evidence and was consistently mentioned by the CTs as a facilitator. Limited knowledge of pain assessment measures, approaches and treatment in neonates hospitalized in NICUs worldwide is consistently described as a barrier to the provision of optimal care by nurses [22–25]. Additionally, nurses and midwives indicate a lack of time to update knowledge and clinical practices related to pain [25]. Thus, readily available and valid implementation strategies are highly valued.

In the *Inner Setting*, *Compatibility,* or how the intervention fits existing workflows and systems, was a salient facilitator noted by the CTs. ImPaC relies on up-to-date, high-quality, synthesized infant pain assessment and treatment evidence, meeting Canadian and global standards [23, 24]. CTs implemented ImPaC without adaptations as it was compatible with local pain practices. Implementing ImPaC to meet infants’ needs [*Patient Needs and Resources*] on improved pain practices was another salient facilitator.

In a web-based survey distributed to NICUs worldwide, 67% of 303 NICUs indicated having local pain management guidelines [26]; however, research continues to demonstrate a significant knowledge to practice gap in procedural pain treatment [3, 27]. ImPaC may play an important role in minimizing this gap by focusing on the QI process (e.g., Plan, Do, Study, Act cycles) of implementing evidence-based information on infant pain at the unit level rather than simply providing evidence-based pain assessment and treatment strategies. This approach acknowledges a required paradigm shift in the responsibility for pain management to organizations rather than solely individuals. Organizations must acknowledge their institutional commitment towards pain prevention and treatment through accountability requirements in their organizational structure, prioritization of key outcomes, fundraising and strategic planning [28, 29].

Lack of *Available Resources*, predominantly time and competing *Relative Priorities*, were salient barriers identified by the CTs, with overlapping reasoning. NICUs are demanding and dynamic environments, and most individuals who joined the CTs did so in addition to rather than part of their current roles (e.g., educator, bedside nurse, advanced practice nursing roles). Some NICUs implemented ImPaC while transitioning from paper to electronic medical records, requiring significant training commitment. These resource issues raise the question of who is best suited for the CT role. Engaging individuals experienced in implementing QI strategies and managing multiple tasks in complex environments may be beneficial.

We also explored the relationships between implementation outcomes and determinants. We found several implementation determinants were associated with feasibility. These constructs included *Champions, Key Stakeholders, Relative Priority* and *Networking and Communications.* These positive associations with feasibility make logical sense in reflecting the availability and willingness to spend time interacting with the website and provide a starting place to tailor local strategies and develop future refinements to ImPaC to enhance uptake and implementation. We found fewer correlations between implementation determinants and intervention fidelity, including statistically significant associations between fidelity and *Engaging Key Stakeholders, Relative Priority, and Patient Needs and Resources*. Associations may be limited due to the small sample size of participating NICUs.

### Strengths and limitations

The implementation process embedded in ImPaC steps was considered logical and easy to follow and navigate. Key implementation strategies such as identifying and preparing the CT (champions), assessing readiness, identifying local barriers and facilitators, audit and feedback, distributing educational materials, making training dynamic, reminding clinicians, and re-examining the implementation process [30], were considered helpful in guiding pain practice change and may be a template for other practice changes. The seven-step approach provided CTs a structured facilitative process for individuals unfamiliar with QI initiatives. ImPaC is based on years of research on pain in neonates, infants, and children [10, 12, 13, 15] with the collaboration of knowledge users and an interdisciplinary group of researchers. Users' collective expertise and consideration were central to CTs, as noted in Table 2. These attributes may have enabled some of the salient facilitators identified, such as *Implementation Climate*, *Engaging Key Stakeholders*, *Reflecting and Evaluating*, and *Patient Needs and Resources*. However, we expect that the user-centered design approach and series of usability testing which preceded this work, combined with key attributes of ImPaC were noted as facilitators, including *Design Quality and Packaging* and *Complexity*, allow for HCPs at any level of their career to be able to navigate through and implement ImPaC.

Fidelity was operationalized as adherence to completing all ImPaC’s steps. Although we consider the 7-step cycle progression as logical and theoretically based, participants indicated that sometimes they continued implementing the KT tools over time, without completing the final planning step (Step 7) or going back to ImPaC and formally completing earlier steps (e.g., Steps 3 to 6). This approach might indicate how ImPaC can be adapted to meet local needs; however, the impact on fidelity and sustainability over time and in maintaining clinical outcomes needs to be further investigated.

The COVID-19 pandemic was a commonly reported barrier to implementing ImPaC and the overall conduct of the study. This finding aligns with *Critical Incidents*, a new construct in the updated CFIR 2.0 framework [30] to include events such as pandemics, weather-related disasters, or political disruptions as ‘large-scale and/or unanticipated events disrupting innovation implementation and/or delivery. The COVID-19 pandemic interrupted research activities for about 3 months in 11 of the 23 participating sites, with many units needing to wait until the pandemic was over before starting implementation. In another setting in Italy, a survey conducted in seven level 3 and six level 2 NICUs revealed that despite the low direct clinical impact of COVID-19 in newborns, clinicians displayed clear signs of mental health load outcomes due to living in a situation of uncertainty and personal exposure to contacts with parents and other relatives of the newborns, and having to carry out activities once routine and now fraught with uncertainty [31].

Pain in preterm and sick infants hospitalized in the NICU is a global concern in terms of both immediate and long-term outcomes. Safe and effective interventions are needed to foster practice change on a larger scale. We are limited in using ImPaC in English-speaking NICUs in one high-income country; thus, cultural relevance to other settings that speak different languages, have different health care systems and are in various income brackets is unknown. We are currently updating ImPaC based on user input so that the tool is living and dynamic. In addition, we will address accessibility and cultural adaptation for a broader spectrum of NICUs. We will also consider the CFIR 2.0 version to include additional constructs and subconstructs for evaluation in the future, hoping to understand better determinants that can be tailored for better results.

## Conclusion and implications

To our knowledge, ImPaC is the first multifaceted web-based implementation strategy designed to facilitate pain practice change in NICUs. We have created a web-based implementation strategy for HCPs caring for hospitalized infants. Our goal is to facilitate the implementation of evidence-based knowledge in a manner that allows for ease of practice change. NICU implementers of ImPaC have varying degrees of IS experience and understanding, requiring a simple and feasible process to guide the practice change work. Identifying barriers and facilitators to implementing ImPaC enables tailoring at the local level to promote clinical and implementation outcomes.

## Supplementary Information

Additional file 1. Detailed criteria used to assign ratings to constructs.

## Data Availability

The data that support the findings of this study are available from the corresponding author, upon reasonable request.

## Abbreviations

CFIR: Consolidated Framework for Implementation Research
COVID-19: Coronavirus disease of 2019
cRCT: Cluster randomized controlled trial
CT: Change team
EPIQ: Evidence-based Practice for Improving Quality
FG: Focus group
HCP: Healthcare professionals
ImPaC: Implementation of Infant Pain Practice Change Resource
INT: Intervention
NICUs: Neonatal Intensive Care Units
QI: Quality Improvement
StaRI: Standards for Reporting Implementation Studies
UC: Usual Care
IS: Implementation Science

## References

[1] Chambers CT. From evidence to influence: dissemination and implementation of scientific knowledge for improved pain research and management. Pain. 2018;159(1):S56-64. 30113948 10.1097/j.pain.0000000000001327

[2] Eccleston C, Fisher E, Howard RF, Slater R, Forgeron P, Palermo TM, et al. Delivering transformative action in paediatric pain: a Lancet Child & Adolescent Health Commission. Lancet Child Adolesc Health. 2021;5(1):47–87. 33064998 10.1016/S2352-4642(20)30277-7

[3] Bueno M, Rao M, Aujla P, Victor C, Stevens B. A scoping review of the epidemiology and treatment of painful procedures in hospitalized neonates: What has changed in the past three decades? Eur J Pain. 2024;28(9):1468–85. 38873730 10.1002/ejp.2294

[4] Anne RP, Deshabhotla S, Ahmed SW, Ahmed SJ, Reddy N, Farooqui D, et al. A quality improvement initiative to improve management of procedural pain in preterm neonates. Paediatr Anaesth. 2021;31(2):221–9. 33188650 10.1111/pan.14075

[5] Klunk CJ, Barrett RE, Peterec SM, Blythe E, Brockett R, Kenney M, et al. An initiative to decrease laboratory testing in a NICU. Pediatrics. 2021;148(1):1–10. 10.1542/peds.2020-000570 34088759

[6] Sawleshwarkar K, Singh M, Bajaj R, Loya S, Chikhlondhe R, Bhave S. Implementing use of sucrose analgesia (non-pharmacological management of neonatal pain) in a standalone private facility level 3 neonatal care unit using point of care quality improvement methodology. BMJ Open Qual. 2022;11:1–7. 10.1136/bmjoq-2022-001830 PMC9204459 35705258

[7] Lyngstad LT, Steinnes S, Le Marechal F. Improving pain management in a neonatal intensive care unit with single-family room-A quality improvement project. Paediatr Neonatal Pain. 2022;4(2):69–7. 10.1002/pne2.12075. 10.1002/pne2.12075 PMC9189914 35719218

[8] Reddy S, Nesargi SV, Stevens S, Jose J, Babu H. Procedural analgesia in the neonatal intensive care unit: a quality improvement initiative. Am J Perinatol. 2022;39(15):1688–92. 33706395 10.1055/s-0041-1726121

[9] Stevens B, Bueno M, Barwick M, Campbell-Yeo M, Chambers C, Estabrooks C, et al. The implementation of infant pain practice change resource to improve infant procedural pain practices: a hybrid type 1 effectiveness-implementation study. Pain. 2024. 10.1097/j.pain.0000000000003496. 39679622 10.1097/j.pain.0000000000003496 PMC12168804

[10] Stevens BJ, Yamada J, Estabrooks CA, Stinson J, Campbell F, Scott SD, et al. Pain in hospitalized children: Effect of a multidimensional knowledge translation strategy on pain process and clinical outcomes. Pain. 2014 [cited 2018 May 14];155(1):60–8. Available from: http://content.wkhealth.com/linkback/openurl?sid=WKPTLP:landingpage&an=00006396-201401000-00013 10.1016/j.pain.2013.09.007 24021861

[11] Widger K, Stevens B, Barwick M. Stories from the floor: a knowledge translation casebook on improving pediatric pain practices. Toronto: CIHR Team in Children’s Pain; 2013. Available from: https://www.sickkids.ca/pdfs/Research/stevens-research/53075-Stories-from-the-floor.pdf

[12] Stevens BJ, Yamada J, Promislow S, Stinson J, Harrison D, Victor JC, et al. Implementation of multidimensional knowledge translation strategies to improve procedural pain in hospitalized children. Implement Sci. 2014 [cited 2018 May 14];9(1):120. 10.1186/s13012-014-0120-1 PMC4263210 25928349

[13] Bueno M, Stevens B, Rao M, Riahi S, Lanese A, Li SA, et al. Usability, acceptability, and feasibility of the Implementation of Infant Pain Practice Change (ImPaC) Resource. Paediatr Neonatal Pain. 2020 [cited 2024 Mar 5];2(3):82–92. 10.1002/pne2.12027 PMC8975234 35547024

[14] Damschroder LJ, Aron DC, Keith RE, Kirsh SR, Alexander JA, Lowery JC. Fostering implementation of health services research findings into practice: a consolidated framework for advancing implementation science. Implement Sci. 2009 [cited 2018 Aug 28];4(1):50. 10.1186/1748-5908-4-50 PMC2736161 19664226

[15] Bueno M, Stevens B, Barwick MA, Riahi S, Li SA, Lanese A, et al. A cluster randomized clinical trial to evaluate the effectiveness of the Implementation of Infant Pain Practice Change (ImPaC) Resource to improve pain practices in hospitalized infants: a study protocol. Trials. 2020;21(1):16. 10.1186/s13063-019-3782-9 PMC6945403 31907017

[16] Proudfoot K. Inductive/Deductive Hybrid Thematic Analysis in Mixed Methods Research. J Mix Methods Res. 2023;17(3):308–26.

[17] Damschroder LJ, Lowery JC. Evaluation of a large-scale weight management program using the consolidated framework for implementation research (CFIR). Implement Sci. 2013;8(1):1–17. 23663819 10.1186/1748-5908-8-51 PMC3656778

[18] CFIR Research Group. CFIR Tools and Templates. 2014 [cited 2024 Dec 11]. Available from: https://cfirguide.org/tools/tools-and-templates/

[19] Husain A, Cohen E, Dubrowski R, Jamieson T, Kurahashi AM, Lokuge B, et al. A Clinical Communication Tool (Loop) for Team-Based Care in Pediatric and Adult Care Settings: Hybrid Mixed Methods Implementation Study. J Med Internet Res. 2021;23(3):e25505. 10.2196/25505 PMC8294640 33656445

[20] Pinnock H, Barwick M, Carpenter CR, Eldridge S, Grandes G, Griffiths CJ, et al. Standards for reporting implementation studies (StaRI): explanation and elaboration document. BMJ Open. 2017;7:13318. 10.1136/bmjopen-2016-013318 PMC5387970 28373250

[21] Trinkley KE, Glasgow RE, D'Mello S, Fort MP, Ford B, Rabin BA. The iPRISM webtool: an interactive tool to pragmatically guide the iterative use of the Practical, Robust Implementation and Sustainability Model in public health and clinical settings. Implement Sci Commun. 2023;4(1):116. 10.1186/s43058-023-00494-4 PMC10508024 37726860

[22] Alotaibi K, Higgins I, Day J, Chan S. Paediatric pain management: knowledge, attitudes, barriers and facilitators among nurses – integrative review. Int Nurs Rev. 2018;65(4):524–33. 29956310 10.1111/inr.12465

[23] Hu J, Ruan H, Li Q, Gifford W, Zhou Y, Yu L, et al. Barriers and facilitators to effective procedural pain treatments for pediatric patients in the Chinese context: a qualitative descriptive study. J Pediatr Nurs. 2020;54:78–85. 32585541 10.1016/j.pedn.2020.06.004

[24] Mala O, Forster EM, Kain VJ. Neonatal Nurse and Midwife Competence Regarding Pain Management in Neonates A Systematic Review. Vol. 22, Advances in Neonatal Care. Lippincott Williams and Wilkins; 2022. pp. E34–42. 10.1097/ANC.0000000000000911 34224481

[25] Mala O, Forster EM, Kain VJ. Thai nurses’ and midwives’ perceptions regarding barriers, facilitators, and competence in neonatal pain management. Adv Neonatal Care. 2024;24(2):E26-38. 38096446 10.1097/ANC.0000000000001128

[26] Ullsten A, Beken S, Campbell-Yeo M, Cavallaro G, Decembrino N, Durrmeyer X, et al. Parents in Neonatal Pain Management—An International Survey of Parent-Delivered Interventions and Parental Pain Assessment. Children. 2024;11(9):1105. 39334637 10.3390/children11091105 PMC11430199

[27] Cruz MD, Fernandes AM, Oliveira CR. Epidemiology of painful procedures performed in neonates: A systematic review of observational studies. European Journal of Pain. 2016 Apr 29 [cited 2018 Aug 28];20(4):489–98. 10.1002/ejp.757 26223408

[28] Health Standards Organization. Pediatric Pain Management. Ottawa Canada: Health Standards Organization, 2023.

[29] Solutions for Kids in Pain. Procedural pain management in children & youth: A toolkit for health professionals, 2023.

[30] Powell BJ, Waltz TJ, Chinman MJ, Damschroder LJ, Smith JL, Matthieu MM, et al. A refined compilation of implementation strategies: results from the Expert Recommendations for Implementing Change (ERIC) project. Implementation Science. 2015;10(1):21. 25889199 10.1186/s13012-015-0209-1 PMC4328074

[31] Gagliardi L, Grumi S, Gentile M, Cacciavellani R, Placidi G, Vaccaro A, et al. The COVID-related mental health load of neonatal healthcare professionals: a multicenter study in Italy. Ital J Pediatr. 2022;48(1):136. 35907872 10.1186/s13052-022-01305-7 PMC9338560

