# Supplementary material for: Enhancing infant pain assessment and treatment: investigating barriers, facilitators, and implementation outcomes with the ImPaC Resource
Source: Implement Sci Commun. 2026 Jan 10;7:25. doi: 10.1186/s43058-026-00856-8 (PMC12882200; doi:10.1186/s43058-026-00856-8)
Supplement: Supplementary file 1 — Additional file 1. Detailed criteria used to assign ratings to constructs. [file 43058_2026_856_MOESM1_ESM.docx]

**Additional file 1** – Detailed criteria used to assign ratings to constructs.

| **Rating** | **Criteria** |
| --- | --- |
| -2 | The construct is a negative influence in the organization, an impeding influence in work processes, and/or an impeding influence in implementation efforts. The interviewees provide detail and describe explicit examples of how the key or all aspects (or the absence) of a construct manifests itself in a negative way.   - Strength of valence is based on amount of detail provided, not on number of interviewees providing examples as focus groups contained different number of participants across sites |
| -1 | The construct is a negative influence in the organization, an impeding influence in work processes, and/or an impeding influence in implementation efforts. Interviewees make general statements about the construct manifesting in a negative way but without concrete examples: |
|  | - The construct is mentioned only in passing or at a high level without examples or evidence of actual, concrete descriptions of how that construct manifests; |
|  | - There is mixed effect of different aspects of the construct but with a general overall negative effect; |
|  | - There is sufficient information to make an indirect inference about the generally negative influence; and/or |
|  | - Judged as weakly negative by the absence of the construct. |
| 0 | A construct has a neutral influence if: |
|  | - It appears to have neutral effect (purely descriptive) or is only mentioned generically without valence; |
|  | - There is no evidence of positive or negative influence; |
| X | The construct has mixed influence if: |
|  | - There are positive and negative influences at different levels in the organization that balance each other out; and/or different aspects of the construct have positive influence while others have negative influence and overall, the effect is neutral. |
| +1 | The construct is a positive influence in the organization, a facilitating influence in work processes, and/or a facilitating influence in implementation efforts. Interviewees make general statements about the construct manifesting in a positive way but without concrete examples |
|  | - The construct is mentioned only in passing or at a high level without examples or evidence of actual, concrete descriptions of how that construct manifests; |
|  | - There is a mixed effect of different aspects of the construct but with a general overall positive effect; and/or |
|  | - There is sufficient information to make an indirect inference about the generally positive influence. |
| +2 | The construct is a positive influence in the organization, a facilitating influence in work processes, and/or a facilitating influence in implementation efforts. Interviewees provide detail and describe explicit examples of how the key or all aspects of a construct manifests itself in a positive way.   - For example: Adaptability: Rate as a +2 if they make explicit examples of how they adapted the Resource into another context. For example, if they used ImPaC in the PICU |
| The * indicates that although there are more positive/negative comments stated and therefore received that rating, there were opposite comments noted. | |
